# The Efficacy of an Active Medicinal Alkaloid, Berbamine, in Reducing Overactive Bladder Symptoms in a Retinyl Acetate-Induced Model

**DOI:** 10.3390/biom15020190

**Published:** 2025-01-29

**Authors:** Jan Wróbel, Łukasz Zapała, Grzegorz Niemczyk, Ewa Poleszak, Piotr Dobrowolski, Tomasz Kluz, Anna Bogaczyk, Patryk Jasielski, Artur Wdowiak, Iwona Bojar, Marcin Misiek, Andrzej Wróbel

**Affiliations:** 1 Medical Faculty, Medical University of Lublin, 20-093 Lublin, Poland; 2 Clinic of General, Oncological and Functional Urology, Medical University of Warsaw, Lindleya 4, 02-005 Warsaw, Poland; grzegorz.niemczyk@wum.edu.pl; 3 Laboratory of Preclinical Testing, Chair and Department of Applied and Social Pharmacy, Medical University of Lublin, 1 Chodźki Street, 20-093 Lublin, Poland; ewapoleszak@umlub.pl; 4 Department of Functional Anatomy and Cytobiology, Faculty of Biology and Biotechnology, Maria Curie-Sklodowska University, Akademicka St. 19, 20-033 Lublin, Poland; piotr.dobrowolski@mail.umcs.pl; 5 Department of Gynecology, Gynecology Oncology and Obstetrics, Institute of Medical Sciences, Medical College of Rzeszow University, Rejtana 16c, 35-959 Rzeszow, Poland; jtkluz@interia.pl; 6 Department of Gynecology, Gynecology Oncology and Obstetrics, Fryderyk Chopin University Hospital, Szopena 2, 35-055 Rzeszow, Poland; annabogaczyk@interia.pl (A.B.); patryk.jasielski111@gmail.com (P.J.); 7 Obstetrics and Gynecology, Faculty of Health Sciences, Medical University of Lublin, 4-6 Staszica St., 20-081 Lublin, Poland; wdowiakartur@gmail.com; 8 Department of Women’s Health, Institute of Rural Health in Lublin, ul. Jaczewskiego 2, 20-090 Lublin, Poland; iwonabojar75@gmail.com; 9 Department of Gynecologic Oncology, Holy Cross Cancer Center, 25-377 Kielce, Poland; marcin.misiek@onkol.kielce.pl; 10 Second Department of Gynecology, Medical University of Lublin, Jaczewskiego 8, 20-090 Lublin, Poland; wrobelandrzej@yahoo.com

**Keywords:** overactive bladder animal model, berbamine, herbal drugs

## Abstract

We aimed to determine whether berbamine (BBM) would have an effect on retinyl acetate (RA)-induced cystometric and biochemical parameters, characteristic of bladder overactivity. BBM exhibits anti-inflammatory, anti-oxidant, and muscle-relaxant effects which could counteract pathophysiological mechanisms observed in overactive bladder (OAB) syndrome. The cohort of 60 rats was divided into 4 groups: I—control, II—RA group, III—BBM, and IV—group with the combination of RA + BBM. The cystometry, BBF, cardiovascular parameters and diuresis, the analysis of the cFos, and biochemical biomarker levels were analyzed 48 h after completion of BRB administration. The examined substance turned out to reverse the cystometric changes and c-Fos expression changes induced by RA when compared to the control group. There were no significant changes observed in the analyzed groups of animals MAP, HR, BBF, or UP. Importantly, BBM also turned out to reduce the level of OAB biomarkers present in urine (NGF, BDNF), urothelium (TRPV1, SNAP29, ATP, CGRP, or OCT-3), bladder detrusor muscle (VAChT, Rho kinase) as well as to reduce the exponents of oxidative stress (3-nitrotyrosine, malondialdehyde). The multifactorial explanation of the successful alleviation of the RA-induced detrusor overactivity makes the concept of incorporation of BBM in the OAB treatment promising for the future research.

## 1. Introduction

Overactive bladder (OAB) is a chronic and bothersome disease with an expected prevalence of 11.8% according to EPIC study [1]. OAB affects health-related quality of life and is thought to be linked with depression and anxiety [2]. Despite being major clinical concern, there are limited pharmacological options for OAB therapy. First-line treatment based on antimuscarinics and, recently, beta3-mimetics, is widely prescribed as a pharmacological treatment for OAB [3]. However, the success perceived as a patient’s reported quality of life may be sometimes hard to obtain. One should also be aware of possible side effects lowering the compliance significantly [4,5]. It is then thought to be the top reason for the discontinuation of the medical treatment and poor compliance within the first 3 months [4]. Finally, the costs of the long-term drug therapy are the other side of the coin. For these reasons, the development of novel pharmacological therapies, along with deep insight into herbal compounds is awaited [6].

Berbamine (BBM) represents one of the bioactive ingredients derived from an old herbal medicine, *Berberis vulgaris* L. It has drawn a lot of attention due to recent findings on its prominent anti-inflammatory activity [7,8]. It is of paramount importance as both inflammation and oxidative stress are implicated in OAB pathophysiology [9]. It is thought to suppresses PI3K, COX-2, phosphorylated-Akt/Akt, and mTOR, while activating p53 [10,11]. Berbamine should be distinguished from berberine, which are both derived from barberry species [12]. Both alkaloids also exhibit anticancer activity. Berbamine is thought to have the potential to influence multiple signaling cascades in cancers, including JAK/STAT [13]. As for the other potential pathways, BRB exerted anticancer activity via the ROS/NF-κB signaling pathway [14], while oxide–alginate–berbamine nanocomposites suppressed the tumor growth and inhibited the PI3K/Akt pathway [15].

Although there are no data on the direct influence of BBM on bladder function, E–6-berbamine, which is a derivative of BBM, was demonstrated in in vitro studies to inhibit α3- and partially α7-nicotinic acetylocholine receptors [16], which are thought to significantly block bladder contractions [17]. Thus, we hypothesized that BBM may be a promising natural agent to be investigated as a potential candidate of bothersome OAB symptoms.

Recently, we have presented that detrusor overactivity (DO) may be experimentally induced by retinyl acetate (RA) in rats [18], and successfully alleviated by specific herbal extracts e.g., *Potentilla chinensis* [19]. Taking into account anti-inflammatory, anti-oxidant, and muscle relaxant properties of BBM, it seems to be a promising agent in further research in the context of OAB treatment. The aim of the current paper was to discover whether BBM would alleviate RA-induced effects in several cystometric and biochemical parameters typical to bladder overactivity, and therefore to find out if this natural compound may become an objective tool to become a pharmacological treatment of OAB patients.

## 2. Materials and Methods

### 2.1. Animals

A total of 60 female Wistar rats (at 250 g) were included in the respective studies. Animals were placed separately in the metabolic cages under standard conditions, as described previously [18]. The animals were assigned to the 4 consecutive groups consisting of 15 animals each at random:

- a group of animals that was administered with saline (the control group, CON)
- a group of animals that was administered with retinyl acetate (RA)
- a group of animals that was administered with berbamine (BBM; 25 mg/kg/day i.p.)
- a group that received of animals that was administered with retinyl acetate plus berbamine (RA + BBM).

### 2.2. Drugs

The following drugs were administered to the animals in the experiments:

- Retinyl acetate (Sigma-Aldrich Fluka, St. Louis, MO, USA): diluted to 0.75% solution with a mixture of Polysorbate 80 and saline and administered intravesically due to the bladder detrusor overactivity induction.
- Berbamine (BBM, MedChem Express, NJ, USA): after dissolution in physiological saline, it was administered for 14 days as an intraperitoneal injection at a daily dosage of 25 mg/kg [18,20,21]. The control group received an analogous volume (10 mL/kg) of physiological saline.

### 2.3. Surgical Procedures

All the surgical experiments were conducted as described elsewhere [18,22], under anesthesia (75 mg/kg of ketamine hydrochloride plus 15 mg/kg of xylazine i.p.).

#### 2.3.1. Conscious Cystometry

The conscious cystometry was described previously [18,22]. Briefly, animals were placed in the supine position. Cefazolin sodium hydrate (Biofazolin, Sandoz, Lublin, Poland) s.c was used in the prophylaxis. Either RA solution or vehicle was applied intravesically for 5 min and then removed again with catheter. The prepared abdominal wall was opened via midline incision. A double lumen catheter was inserted into the apex of the bladder and sutured, while the abdomen was closed in multiple layers. Cystometric studies were performed 16 days after the surgical procedures (i.e., 2 days after the last dose of BBM), as previously established in the previous papers [18,22]. The bladder was filled using microinjection pump with physiological saline (at 0.05 mL/min rate, RT) to induce repetitive voiding at the rate of 0.05 to 0.1 mL/min. Cystometry profiles and micturition volumes were displayed on a Grass polygraph. The data were analyzed using a sampling rate of 10 samples/s. The measurements in each animal represent the average of 5 bladder micturition cycles after eliciting repetitive voiding. The mean from all rats in each condition were averaged to create pooled data for each condition. The following abbreviations for cystometric parameters were used: BP—basal pressure (cm H_2_O); TP—threshold pressure (cm H_2_O); MVP—micturition voiding pressure (cm H_2_O); VV—voided volume (mL); PVR—post-void residual (mL); ICI—intercontraction interval (s); DOI—detrusor overactivity index (cm H_2_O/mL); FNVC—non-voiding contractions frequency (times/filling phase); VTNVC—volume threshold to elicit NVC (%); ANVC—nonvoiding contractions amplitude (cm H_2_O); BC—bladder compliance (mL/cm H_2_O); AUC—the area under the pressure curve (cm H_2_O/s).

#### 2.3.2. Bladder Urothelium Tissue and Detrusor Muscle Preparation

The bladder was excised at the level of the proximal urethra, as described by Park et al. [23]. The urothelium was dissected from the bladder detrusor muscle under a microscope by injections with physiological saline via a microinjection pump (CMA 100; Microject, Solna, Sweden).

### 2.4. Bladder Blood Flow (BBF)

The BBF (repeated 5 times for each rat with empty bladder) was analyzed by laser Doppler blood perfusion imager (PeriScan PIM III, Perimed, Stockholm, Sweden) and presented as changes in the laser Doppler frequency by a color scale.

### 2.5. The Assessment of Cardiovascular Parameters and Diuresis

After the cystometry, the animals remained separate in the cages for 24 h and the influence of BBM on mean arterial pressure (MAP), heart rate (HR), and daily urine production (UP) was determined.

### 2.6. Determining the Expression Levels of cFos in Central Micturition Areas

Based on the stereotactic atlas of the rat’s brain and the bregma data, the PMC, vlPAG, and MPA were isolated [24]. There were 10 sections on average per region collected from each animal. C-Fos (c-Fos; MyBioSource, MBS729725) expression was measured in the respective central micturition areas: medial preoptic area (MPA), the ventrolateral periaqueductal gray (vlPAG), and pontine micturition center (PMC).

### 2.7. Biochemical Analyses

The assessment of biochemical changes was performed using ELISA kits, analyzing the level of biomarkers characteristic for OAB in urothelium (CGRP, TRPV1, OCT3, ATP, and SNAP-29), the detrusor muscle (VAChT, Rho Kinase), or urine (BDNF, NGF). Additionally, the level of oxidative stress markers (MAL, 3NIT) was determined in urothelium. In order to standardize the results, the level of the assessed biochemical parameters was given in pg/mL.

### 2.8. The Research Project

After the implantation of catheters into the bladder and carotid artery, the animals received BRB or 0.9% NaCl for 14 consecutive days. Cystometric measurements and BBF were performed in conscious animals 48 h after the last injection. Then, animals were placed in cages for one day to assess diuresis, blood pressure, and activity of the cardiac impulse–conduction system. An analysis of changes in biochemical parameters was performed immediately after decapitation.

### 2.9. Statistics

The obtained results were evaluated using the Bonferroni test, which was preceded by two-way analysis of variance. The results with a *p* value of <0.05 were considered statistically significant.

All the applied procedures were approved by the Local Ethics Committee and they were performed in accordance with binding European law related to the experimental studies on animal models.

## 3. Results

### 3.1. The Effects of BBM on RA-Induced Changes in the Urodynamic Parameters

**Figure 1:**
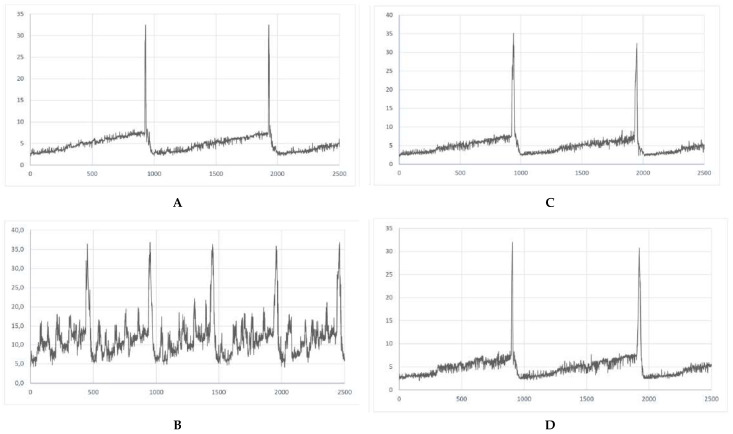
Representative cystometric traces for each experimental group. (**A**) Cystometrogram in control rats (CON); (**B**) cystometrogram in rats that received retinyl acetate (RA); (**C**) cystometrogram in rats that received berbamine (BBM); (**D**) cystometrogram in rats that received retinyl acetate plus berbamine (RA + BBM).

RA was found to influence the urodynamic parameters that are analogous to those reported in patients with detrusor overactivity. A statistically significant increase in BP, TP, DOI, FNVC, VTNVC, and bladder compliance was demonstrated (Table 1). Furthermore, RA initiated several changes in the voiding phase, such as decrease in VV and ICI while increased AUC (Table 1). Interestingly, in the group in which we administered the combination of RA and BBM, a decrease in BP, DOI, FNVC, ANVC, and AUC was noted, when collated to the RA group. What is more, in the combination group the following urodynamic parameters were increased: TP, VV, ICI, VTNC, and BC, being the sign of the successful reversal of symptoms induced by RA. A representative cystometric traces for each group were provided to illustrate the baseline and experimental findings clearly (Figure 1).

### 3.2. The Effects of BBM on RA-Induced Changes in the BBF

**Figure 2:**
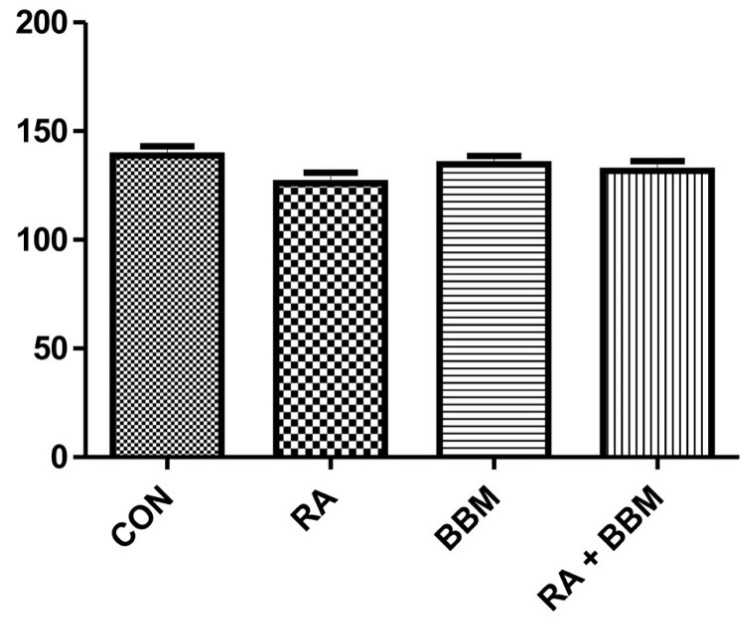
The effects BBM on RA-induced changes in BBF.

We noted no notable changes in the BBF in the experiments (Figure 2).

### 3.3. The Effects of BBM on RA-Induced Changes in the Cardiovascular Parameters and Diuresis

When analyzing further the influence of BBM on basic cardiovascular values and UP, we failed to prove any significant changes (Table 2).

### 3.4. The Effects of BBM on RA-Induced Changes in the Expression Levels of c-Fos in Central Micturition Areas

**Figure 3:**
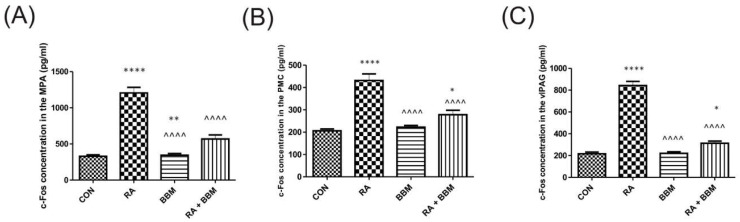
The effects of BBM on c-Fos expression [pg/mL) in the neuronal voiding centers: (**A**) MPA, (**B**) PMC, and (**C**) vlPAG. Abbreviations: control group (CON), RA group (RA), BBM only group (BBM), and RA group treated with BBM (RA + BBM). * *p* < 0.05; ** *p* < 0.01; **** or ^^^^ *p* < 0.0001. * significantly different from the control group. ^ significantly different from the RA group. One-way ANOVA: for MPA: F (3.56) = 66, *p* < 0.0001; for PMC: F (3.56) = 30, *p* < 0.0001; for vlPAG: F (3.56) = 156, *p* < 0.0001.

In animals subjected to RA instillation, a notable increase in c-Fos values was observed in all analyzed voiding centers, which normalized after BBM application, reaching the values observed in the control group (Figure 3).

### 3.5. The Effects of BBM on RA-Induced Changes in the Biochemical Analyses of Biomarkers in Urine

**Figure 4:**
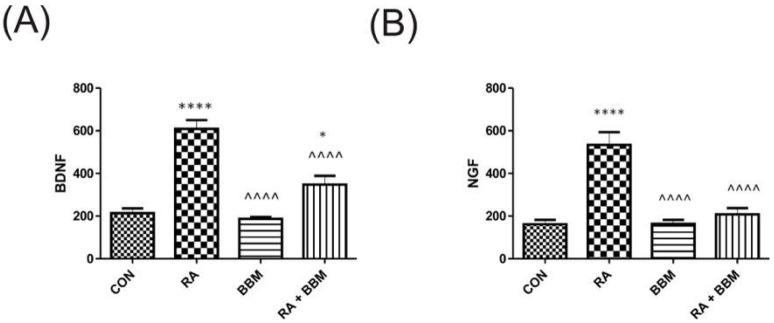
Changes in the biomarkers’ concentrations in urine in the analyzed groups of animals: (**A**) BDNF, and (**B**) NGF. * *p* < 0.05; **** or ^^^^ *p* < 0.0001. * significantly different from the control group. ^ significantly different from the RA group. One-way ANOVA: for BDNF: F (3.56) = 37, *p* < 0.0001, and for NGF: F (3.56) = 25, *p* < 0.01.

The elevation of BDNF and NGF analyzed in urine in the RA group was noted when compared to control group (Figure 4). Furthermore, the administration of BBM resulted in a statistically significant decrease in the values of all the above mentioned markers in animals with RA-induced DO.

### 3.6. The Effects of BBM on RA-Induced Changes in the Biochemical Analyses of Biomarkers in the Bladder Detrusor Muscle

**Figure 5:**
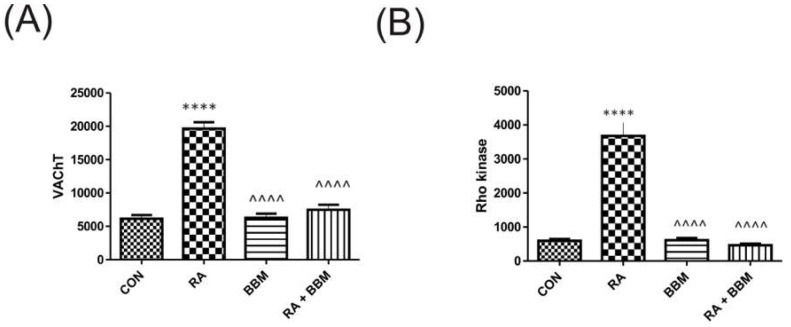
Changes in the biomarkers’ concentrations in bladder detrusor muscle in the analyzed groups of animals: (**A**) VAChT and (**B**) Rho kinase. **** or ^^^^ *p* < 0.0001. * significantly different from the control group. ^ significantly different from the RA group. One-way ANOVA: for VAChT: F (3.56) = 79, *p* < 0.0001, and for Rho kinase: F (3.56) = 42, *p* < 0.0001.

Similarly to the urine biomarkers, RA administration resulted in the respective changes in the biomarkers’ concentration in the bladder detrusor muscle i.e., VAChT and Rho kinase (Figure 5). The administration of the BBM ameliorated those effects.

### 3.7. The Effects of BBM on RA-Induced Changes in the Biochemical Analyses of Biomarkers in the Bladder Urothelium

**Figure 6:**
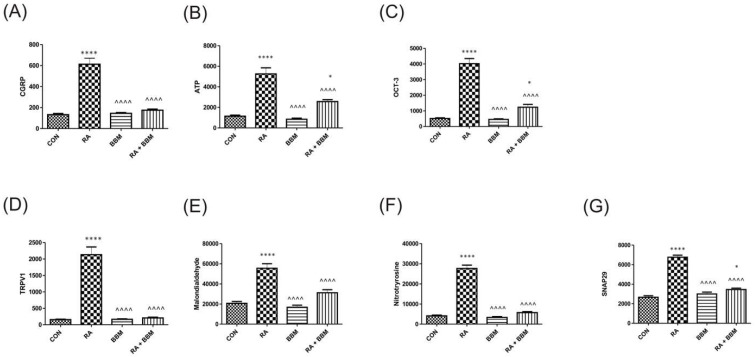
Changes in the biomarkers’ concentrations in bladder urothelium in the analyzed groups of animals: (**A**) CGRP, (**B**) ATP, (**C**) OCT-3, (**D**) TRPV1, (**E**) MAL, (**F**) NIT, (**G**) SNAP29. * or ^ *p* < 0.05; ** or ^^ *p* < 0.01; *** or ^^^ *p* < 0.001, **** or ^^^^ *p* < 0.0001. * significantly different from the control group. ^ significantly different from the RA group. One-way ANOVA: for CGRP F (3.56) = 56, *p* < 0.0001; for ATP F (3.56) = 40, *p* < 0.0001; for OCT-3 F (3.56) = 79, *p* < 0.0001; for TRPV1 F (3.56) = 68, *p* < 0.0001; for MAL F (3.56) = 32, *p* < 0.0001; for NIT F (3.56) = 162, *p* < 0.0001, and for SNAP29 F (3.56) = 115, *p* < 0.0001.

As expected, and proven in other compartments, no notable changes in the analyzed biomarkers after the administration of BBM were observed in comparison with controls (Figure 6A–G). However, RA instillation resulted in an increase in CGRP, ATP, OCT-3, TRPV1, OCT-3, malondialdehyde, 3-nitrotyrosine, and SNAP29. A successful restoration of all these changes in concentrations was observed after administration of BBM (RA + BBM combination group) when compared to control group.

## 4. Discussion

**Figure 7:**
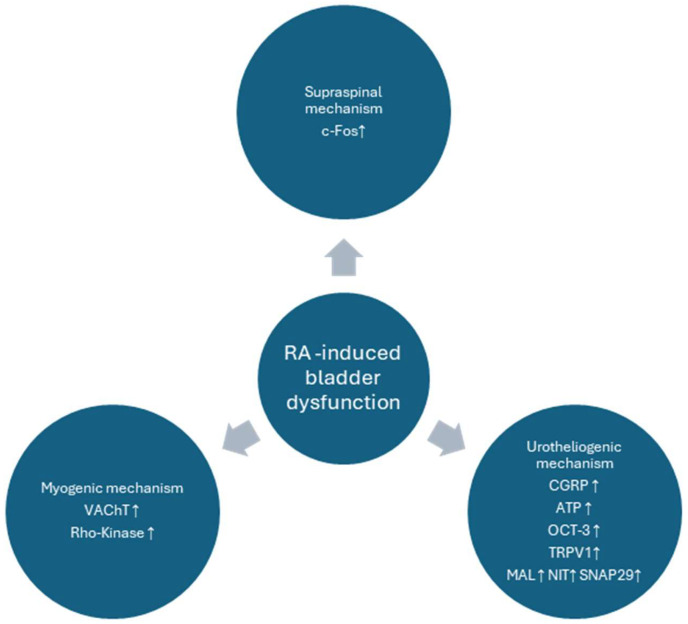
Major mechanisms involved in the pathophysiology of the RA-induced model of bladder overactivity. In our current paper the pathological findings were alleviated to a great extent by BBM. The diagram is based on the paper by Peyronnet et al., modified [26].

Although OAB is diagnosed with clinical symptoms, animal models are based on cystometric findings [25]. In our study, we analyzed a plethora of cystometric parameters, which replicate some of the possible urodynamic findings in OAB patients and, thus, may be of particular importance, when assessing novel candidates for pharmacological applications. Here, we based our study on a well-developed model of animal bladder overactivity, as described previously [18]. Some major findings of the mechanisms were presented in Figure 7, while, interestingly, these were successfully alleviated to a great extent by the influence of BBM. We found that our agent of herbal origin, BBM, initiated the restoration of major urodynamic changes induced by RA in the rat model of DO, i.e., a decrease in BP, DOI, FNVC, ANVC, and AUC, and conversely an increase in TP, VV, ICI, VTNC, and BC were noted.

Here, we have also analyzed BBF, as there is a growing body of evidence indicating that chronic bladder ischemia is among the pathological factors responsible for OAB [27]. RA acts as a TRPV1 agonist and mediates sensory hypersensitivity, which explains the observed RA-induced cystometric parameters regardless of BBF [19]. What is more, our data show that the BBA mechanism of action on bladder function is independent of BBF.

We further analyzed cardiovascular parameters, as there is a link between metabolic syndrome, of which hypertension is one of the components, and OAB [26]. What is more, spontaneous hypertensive rats exhibit DO and are used as animal model of OAB [25]. Systemic BBM administration had no effect on HR and MAP, which claims its cardiovascular safety. Furthermore, in our animal model, no effect on urine production was noted, which clearly indicates the low risk of potential side effects in our experimental configuration. On the other hand, these basic findings shed some light on the preclinical safety of the studied compound. However, intravesical administration of RA has influenced neither MAP nor HR in rats, which excludes those factors as potential triggers for DO. Additionally, we have excluded UP as a factor that could confound our cystometric results.

The lack of effect of intravesical RA administration on BBF and cardiovascular parameters confirms its local mode of action and enable our OAB animal model suitable to study myogenic, urotheliogenic, and neurogenic pathophysiological phenotypes of OAB [26]. Increased c-Fos expression is a well-established marker of neuronal activation [28]. Thus, intravesical RA administration results in the sustained activation of supraspinal neuronal voiding centers i.e., MPA, PMC, and vlPAG. PMC is of particular interest, as it directly controls bladder motoneurons and its excessive stimulation could result in DO [29,30]. It has been observed in our study that BBM prevented the activation of supraspinal neuronal voiding centers. However, direct mechanism of action has to be elucidated, as BBM could possibly exert effect on both receptor, peripheral, and central nervous system.

NGF and BDNF are neutrophins used as biomarkers in OAB [31]. Their overexpression in urine is linked to DO, as OAB patients without DO present with urine levels of both neutrophins within normal range [31]. NGF per se could provoke DO [31]. It may be due to TrkA-mediated activation of TRPV1 in urothelial cells, while TRPV1 and mechanosensory channels were thought to increase expression and sensitization of suburothelial afferent C-fibers [32]. This is consistent with our results as, in rats with RA-induced DO, we observed elevated levels of both NGF and BDNF. Interestingly, the intravesical administration of BBA normalized urine concentrations of both neutrophins and DO. Similarly, in OAB patients, an effective antimuscarinic treatment caused a reduction in NGF concentration in urine [33].

VAChT is a well-established maker of cholinergic neurons, and its increased expression in the detrusor muscle of rats after intravesical RA administration implies increased cholinergic neurotransmission [34,35]. This effect may be, at least partially, explained by BDNF-induced overexpression of VAChT and ChAT, which was demonstrated in a bladder in a rat model [36]. In this context, BBM inhibits cholinergic neurotransmission; however its exact mechanism of action has yet to be determined. RhoA/Rho Kinase pathway, whose activity depends on the stimulation of M2 and M3 receptors, is involved in regulating bladder muscle tone and bladder contractions [37]. Consequently, its increased activity has been implicated in the pathophysiology of involuntary bladder contractions [38]. In our study, Rho kinase expression was significantly increased in the detrusor muscle of rats after intravesical RA administration. Taking into account the decreased cholinergic neurotransmission and activation of M2 and M3 receptors, it could explain BBM-induced normalization of Rho kinase expression in the detrusor muscle of rats after intravesical RA administration.

TRPV1 and CGRP are co-localized in afferent C-fibers extending throughout bladder layers into urothelium [39]. The increased expression of NGF in rats after the intravesical instillation of RA could explain the simultaneous overexpression of TRPV-1 and CGRP observed in our study. CGRP may be responsible for local neuroinflammatory responses and enhance neuron sensory excitability [40]. Therefore, BBM ability to diminish TRPV1 and CGRP expression represents its promising therapeutic properties.

Urothelial cells are capable of the synthesis of acetylcholine and its release through OCT-3 [41]. Such non-neuronal acetylcholine release contributes to mechanoafferent transduction and modulate neural excitability via autocrine and paracrine actions on other urothelial cells, nerves, and smooth muscles, and disruption of this mechanism may be contributing factor to OAB [42]. In our study, an increased OCT-3 expression was observed in the urothelium of rats after the intravesical instillation of RA, which may also imply an increased non-neuronal acetylcholine release.

Elevated ATP concentrations in urine is often found in patients with OAB and is considered one of the markers of the disease [31]. ATP is released from urothelium in response to stretch and activates P2X3 receptors localized in suburothelial afferent neurons facilitating neurotransmission [31]. Additionally, the direct stimulation of muscarinic and TRPV1 receptors, as in our study, may evoke ATP release [43].

Oxidative stress was clearly demonstrated to be associated with OAB [44]. Both malondialdehyde and 3-nitrotyrosine are markers of oxidative stress, as a result of lipid peroxidation and peroxynitrite-mediated nitration of tyrosine, respectively [3,45]. Oxidative damage leads to denervation, as well as fibrosis and apoptosis in the bladder muscle that eventually results in bladder overactivity [44]. Here, we demonstrated BBM to have antioxidative action in rats with detrusor overactivity after the intravesical administration of RA. BBM was proven to activate the Nrf2/ARE signaling pathway responsible for antioxidative machinery [8]. What is more, Nrf2 contribution in OAB pathophysiology was shown in an animal model of OAB, in which Nrf2 activation diminished oxidative stress and improved bladder overactivity [46].

The curative action of botulinum toxin after its internalization into the cytoplasm may be due to the cleavage of specific sites of synaptosomal-associated protein-25 (SNAP-25) and inhibition of the exocytosis of neurotransmitters from the nerve terminals [47]. The SNAP-29 gene, which is a member of the SNAP25 gene family, encodes a protein that plays a role in multiple membrane-trafficking steps [48]. Interestingly, there was an inhibition of SNAP-29 release due to retinyl-induced effects via BBM-mediated mechanisms observed in our study. This may be another phenomenon unraveling BBM ways of action.

As for the major limitations of the study, one should perceive the experiments as in vivo model and should not extrapolate the results into the human population, which necessitates further clinical studies. The details on schedules of the drug administrations were gained in the preliminary unpublished experiments due to little data existing in the literature.

## 5. Conclusions

In the present study, we performed a thorough analysis of a novel candidate for the possible future studies on OAB treatment. The mechanisms involved in BBM action may serve as an adjunct to novel projects on herbal compounds. The multifactorial explanation of the successful alleviation of the RA-induced detrusor overactivity makes the concept of incorporation of BBM in the OAB treatment even more interesting for the future research.

## Figures and Tables

**Table 1:** The effects of BBM on RA-induced changes in the cystometric parameters. * or ^ *p* < 0.05; ** or ^^ *p* < 0.01; *** *p* < 0.001, **** or ^^^^ *p* < 0.0001. * significantly different from the control group. ^ significantly different from the RA group. One-way ANOVA: for BP F (3,56) = 23, *p* < 0.0001; for TP F (3,56) = 11, *p* < 0.0001; for DOI F (3,56) = 43, *p* < 0.0001; for FNVC F (3,56) = 76, *p* < 0.0001; for VTNVC F (3,56) = 19, *p* < 0.0001; for ANVC F (3,56) = 21, *p* < 0.0001; for BC F (3,56) = 7, *p* < 0.001; for MVP F (3,56) = 0.71, NS; for ICI F (3,56) = 19, *p* < 0.0001; for VV F (3,56) = 16, *p* < 0.0001; for PVR F (3,56) = 1.1, NS, and for AUC F (3,56) = 8.7, *p* < 0.0001.

	CONTROL	RA	BBM	RA + BBM
*Storage phase*				
**BP**	3.0 ± 0.62	5.6 ± 1.3 ****	2.9 ± 0.73 ^^^^	3.3 ± 1.3 ^^^^
**TP**	9 ± 2.4	5.3 ± 1.6 ****	8.8 ± 1.9 ^^^^	7.9 ± 2.1 ^^
**DOI**	38 ± 22	320 ± 150 ****	41 ± 17 ^^^^	131 ± 30 *^^^^
**FNVC**	0.41 ± 0.25	4.7 ± 1.6 ****	0.57 ± 0.3 ^^^^	1.3 ± 0.72 *^^^^
**VTNC**	63 ± 14	32 ± 6.7 ****	66 ± 20 ^^^^	45 ± 12 **
**ANVC**	2.8 ± 0.65	5.6 ± 1.5 ****	3.1 ± 0.79 ^^^^	4.3 ± 1.2 **^^
**BC**	0.53 ± 0.17	0.32 ± 0.11 **	0.47 ± 0.11	0.52 ± 0.17 ^^^^
*Voiding phase*				
**MVP**	45 ± 6.5	42 ± 8.8	41 ± 9.5	43 ± 8.9
**ICI**	1110 ± 172	693 ± 159 ***	1089 ± 174 ^^^^	914 ± 181 *^^
**VV**	0.90 ± 0.059	0.57 ± 0.19 ***	0.90 ± 0.13 ^^^^	0.84 ± 0.19 ^^^^
**PVR**	0.078 ± 0.012	0.082 ± 0.012	0.072 ± 0.017	0.078 ± 0.016
**AUC**	16 ± 4.0	23 ± 3.3 ****	18 ± 3.9 ^^	19 ± 4.1 ^

**Table 2:** The effects of the BBM on RA-induced changes in cardiovascular parameters and diuresis.

	CON	RA	BBM	RA + BBM
MAP (mm Hg)	139 ± 14	149 ± 16	136 ± 16	143 ± 8.8
HR (beats/min)	236 ± 19	218 ± 24	222 ± 33	234 ± 30
UP (mL/day)	21 ± 2.7	20 ± 2.7	23 ± 4.0	22 ± 2.6

## Data Availability

The data presented in this study are available on request from the corresponding authors.

## Abbreviations

The following abbreviations are used in this manuscript:

ANVC	nonvoiding contractions amplitude (cm H_2_O)
ATP	adenosine triphosphate (pg/mL)
AUC	the area under the pressure curve (cm H_2_O/s)
BBF	bladder blood flow (color scale)
BBM	berbamine
BC	bladder compliance (mL/cm H_2_O)
BDNF	brain-derived neurotrophic factor (pg/mL)
BP	basal pressure (cm H_2_O)
c-Fos	AP-1 transcription factor subunit (pg/mL)
CGRP	Calcitonin Gene Related Peptide (pg/mL)
DO	detrusor overactivity
DOI	detrusor overactivity index (cm H_2_O/mL)
FNVC	nonvoiding contractions frequency (times/filling phase)
HR	heart rate (beats/min)
ICI	intercontraction interval (s)
MAL	Malondialdehyde (pg/mL)
MAP	arterial pressure (mm Hg)
MPA	medial preoptic area
MVP	micturition voiding pressure (cm H_2_O)
NGF	nerve growth factor (pg/mL)
NIT	3-Nitrotyrosine (pg/mL)
OAB	overactive bladder syndrome
OCT3	Organic Cation Transporter 3 (pg/mL)
PMC	pontine micturition center
PVR	post-void residual (mL)
ROCK	rho kinase (pg/mL)
SNAP-29	Rat Synaptosome Associated Protein 29
TP	threshold pressure (cm H_2_O)
TRPV1	Transient Receptor Potential Cation Channel Subfamily V, Member 1
UP	urine production (mL/day)
VAChT	vesicular acetylcholine transporter (pg/mL)
vlPAG	ventrolateral periaqueductal gray
VTNVC	volume threshold to elicit NVC (%)
VV	voided volume (mL)
